# A novel method for 3D face symmetry reference plane based on weighted Procrustes analysis algorithm

**DOI:** 10.1186/s12903-020-01311-3

**Published:** 2020-11-11

**Authors:** Yujia Zhu, Shengwen Zheng, Guosheng Yang, Xiangling Fu, Ning Xiao, Aonan Wen, Yong Wang, Yijiao Zhao

**Affiliations:** 1 Center of Digital Dentistry, Peking University School and Hospital of Stomatology, No.22 Zhongguancun Avenue South, Haidian District, Beijing, 100081 China; 2 National Engineering Laboratory for Digital and Material Technology of Stomatology, No.22 Zhongguancun Avenue South, Haidian District, Beijing, 100081 China; 3 NHC Key Laboratory of Digital Technology of Stomatology, No.22 Zhongguancun Avenue South, Haidian District, Beijing, 100081 China; 4 Beijing Key Laboratory of Digital Stomatology, No.22 Zhongguancun Avenue South, Haidian District, Beijing, 100081 China; 5 National Clinical Research Center for Oral Diseases, No.22 Zhongguancun Avenue South, Haidian District, Beijing, 100081 China; 6 School of Software Engineering, Beijing University of Posts and Telecommunications, No.10 Xitucheng Road, Haidian District, Beijing, 100876 China; 7 Key Laboratory of Trustworthy Distributed Computing and Service, Ministry of Education, Beijing University of Posts and Telecommunications, No.10 Xitucheng Road, Haidian District, Beijing, 100876 China

**Keywords:** Symmetry reference plane, Procrustes analysis, Three-dimensional facial data, Mandibular deviation, Anatomic landmarks

## Abstract

**Background:**

We aimed to establish a novel method, using the weighted Procrustes analysis (WPA) algorithm, which assigns weight to facial anatomical landmarks, to construct a three-dimensional facial symmetry reference plane (SRP) for mandibular deviation patients.

**Methods:**

Three-dimensional facial SRPs were independently extracted from 15 mandibular deviation patients using both our WPA algorithm and the standard PA algorithm. A reference plane was defined to serve as the ground truth. To determine whether the WPA SRP or the PA SRP was closer to the ground truth, we measured the position error of mirrored landmarks, the facial asymmetry index (FAI) error, and the angle error for the global face and each facial third partition.

**Results:**

The average angle error between the WPA SRP and the ground truth was 1.66 ± 0.81°, which was smaller than that between the PA SRP and the ground truth. The position error of the mirrored landmarks constructed using the WPA algorithm in the global face (3.64 ± 1.53 mm) and each facial partition was lower than that constructed using the PA algorithm. The average FAI error of the WPA SRP was − 7.77 ± 17.02 mm, which was smaller than that of the PA SRP.

**Conclusions:**

This novel automatic algorithm, based on weighted anatomic landmarks, can provide a more adaptable SRP than the standard PA algorithm when applied to severe mandibular deviation patients and can better simulate the diagnosis strategies of clinical experts.

## Background

Mandibular deviation is one of the more common manifestations of facial asymmetry, accounting for 70–80% of all cases [1–3]. The restoration of symmetrical, coordinated, and aesthetic facial shapes is a central focus of oral and maxillofacial surgery, orthodontics and prosthodontics [4–6]. Using three-dimensional digital technology, the extraction of the symmetry reference plane (SRP) is the primary step during symmetry analysis of three-dimensional facial data [7]. SRP accuracy directly affects the symmetry index and is critical for developing treatment strategies and evaluating treatment progress.

The traditional methods for extracting an SRP are often based on medical and bilateral anatomical landmarks measured either using a digital three-dimensional facial model or having the head in a natural position [8–11]. These methods are widely used, but since landmarks definition varies, establishing common methods suitable for different types of facial asymmetry remains challenging [12, 13]. In recent years, an SRP extraction method, referred to as the original-mirror alignment method, based on superimposed three-dimensional original and mirror facial data has received an increasing attention.

This method involves superimposing a three-dimensional geometric shape of a facial model (the original model) onto its mirror model [14]. The SRP of the original model is determined by analysing the superimposed model’s symmetry plane, which are geometrically symmetrical. The most critical step of this method is the three-dimensional superimposition, which primarily involves the iterative closest point (ICP) and Procrustes analysis (PA) algorithms [15, 16]. The ICP algorithm seeks optimal superimposed position of the three-dimensional original and mirror models composed of tens of thousands of point clouds determined by an iterative solution [17]. Based on 1:1 ratio between the original and mirror landmarks (anatomical landmarks or mathematical facial mask), the PA algorithm obtains the superimposed position with minimum average distance between the two sets of landmarks through a matrix operation (translation, rotation, and scale) [18–21]. SRP extraction, using PA algorithm, relies more on facial landmarks than it does when using ICP algorithm. Furthermore, the PA algorithm is more aligned with stomatological clinical diagnosis and treatment and has thus received significant attention in recent years.

Xiong et al. reported that PA algorithm can be used to extract facial SRP using 21 important anatomical landmarks. While this algorithm is suitable for normal facial data, it is not ideal for facial asymmetry data (particularly data from patients with complex facial deformities) since it does not assign weights to individualised facial features (different degrees of asymmetry). There remains a discrepancy between the algorithm results and the logical basis of oral clinical diagnosis [22].

Based on standard PA algorithm studies, this study aims to establish a weighted Procrustes analysis (WPA) algorithm for extracting a three-dimensional facial SRP that can automatically recognise weight assignment of facial landmarks. Our study analysed and evaluated the WPA algorithm suitability for commonly observed clinical cases of mandibular deviation.

## Methods

### Subjects

Fifteen patients from the Department of Oral and Maxillofacial Surgery, Orthodontics and Prosthodontics at the Peking University School and Hospital of Stomatology were recruited for this study. The inclusion criterion was an apparent facial asymmetry with a mandibular deviation of at least 3 mm from the facial midline, which is perpendicular to the interpupillary line at the soft tissue nasion when the patient is seated in a natural head position. The exclusion criteria were a history of previous craniofacial trauma, orthognathic surgery, orthodontic treatment, or congenital anomalies. This study was approved by the bioethics committee of the Peking University School and Hospital of Stomatology (PKUSSIRB-20163113) and was conducted in accordance with the guidelines and regulations for research involving human subjects. All participants were fully informed of the experimental purpose and procedure and provided an informed consent form prior to participating in the study.

### Experimental equipment and software

A Face Scan 3D sensor system (3D-Shape Corp, Germany, Erlangen) was used to collect three-dimensional facial data from each patient, which were obtained in only 0.2–0.8 s with high accuracy (0.1 mm). The scanning range was 270°–320°, the imaging principle was raster scanning using 5 million charge-coupled device (CCD) pixels, and the approximate number of point cloud was 10,000, and 20,000 triangular meshes are formed.

For data processing, we used the reverse engineering software Geomagic Studio 2013 (3D System, USA, Morrisville), which is used to process three-dimensional facial data and conduct SRP extraction. The WPA algorithm developed in this study was based on the Python programming language, which optimises the objective function of the PA algorithm. The PA objective function, F, is shown in formula 1, the weight factor, w_i_, is shown in formula 2, and the WPA objective function F' is shown in formula 3.

1 $$F = \mathop {\min }\limits_{Q} \mathop \sum \limits_{i = 1}^{p}\parallel LMK\_Org_{i} - LMK\_Mir_{i} \parallel_{2}$$

2 $$w_{i} = \frac{1}{{\parallel LMK\_Org_{i} - LMK\_Mir_{i} \parallel_{2} }}$$

3 $$F^{\prime } = \mathop {\min }\limits_{Q} \mathop \sum \limits_{i = 1}^{p} w_{i}\parallel LMK\_Org_{i} - QLMK\_Mir_{i} \parallel_{2}$$

where w_i_ (i = 1,2, …, 32) is the weight factor for each facial landmark (assigned according to the degree of asymmetry of the landmarks), LMK_Org is the original model landmark set, LMK_Mir is the mirror model landmark set, LMK_Org_i_ and LMK_Mir_i_ (i = 1,2, …, n) are the corresponding landmarks in the original and mirror landmark set, respectively, Q is the spatial change matrix (contains translation, rotation, and scale; the scale value is 1 in this study), and p is the number of landmarks.

### Data capturing and processing

When acquiring the three-dimensional facial data, we calibrated the equipment prior to use to ensure accurate image acquisition. Patients were guided by a clinician to a natural head position at distance of 135 cm from the scanner and a sitting position with both eyes looking forward, keeping the Frankfort horizontal (FH) plane parallel to the floor. Data was obtained when the facial expression was naturally relaxed. The criteria for the face scan data were an effective display of facial contours, a high-resolution image, no obvious movement, and a closed mouth.

**Fig. 1:**
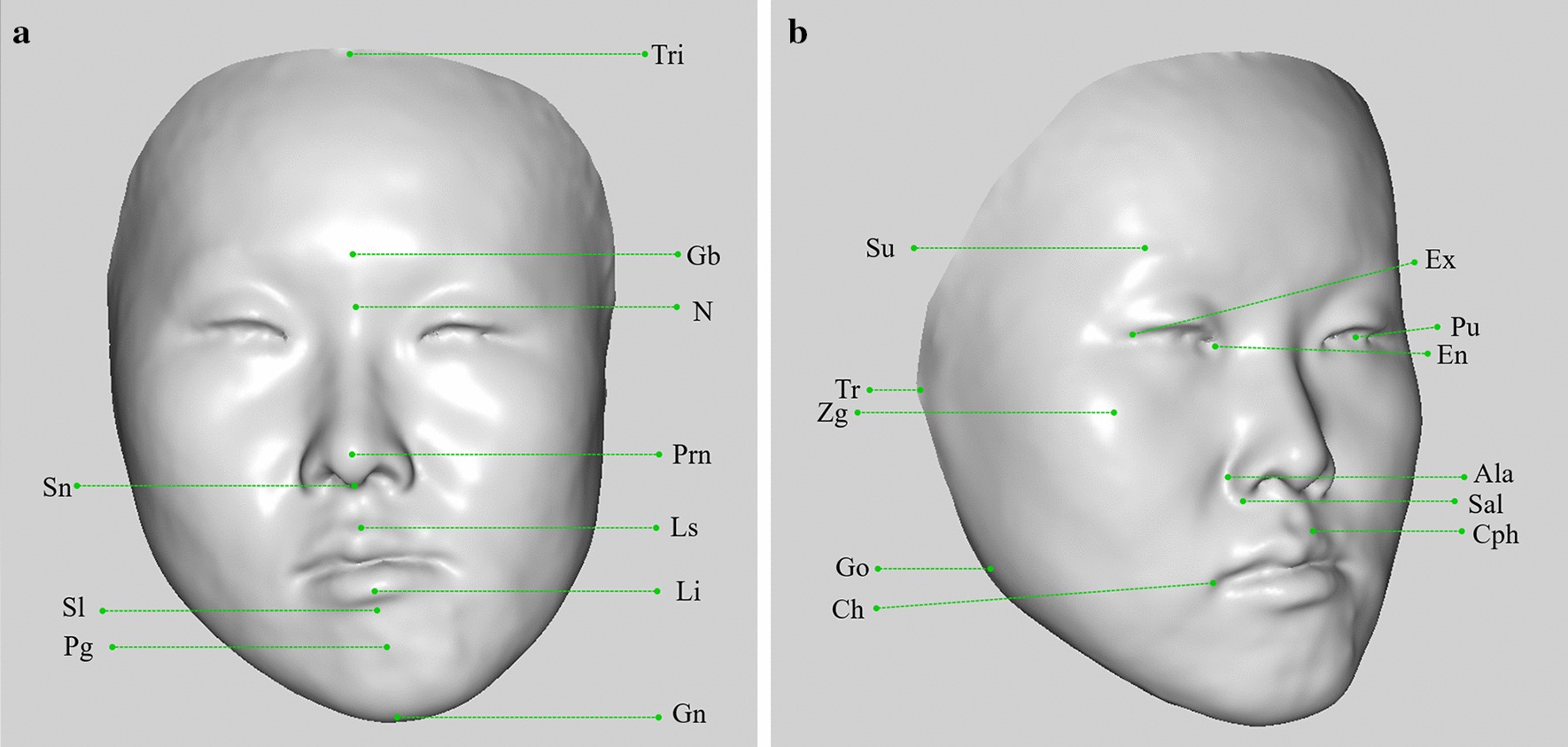
The 32 anatomic landmarks that are used in this study. (Upper facial third: trichion, glabella, superciliary ridge; Middle facial third: nasion, pronasale, subnasale, endocanthion, exocanthion, pupil, alare, subalare, zygion, tragion; Lower facial third: labiale superius, labiale inferius, sublabiale, pogonion, gnathion, cheilion, gonion, crista philtre)

Geomagic Studio 2013 was used to process images, which included removing extra data, smoothing the shells, and filling small holes. The original three-dimensional facial model was adjusted to the natural head position so that the FH plane of the natural head position coincided with the XZ plane of the global coordinate system and the sagittal plane coincided with the YZ plane of the global coordinate system. Three experienced clinical professors completed the extraction of anatomical landmarks from each original facial model (Model_Org). Thirty-two anatomical landmarks were selected from the overall region, including the glabella, nasion, pogonion, and alare et al. An example of a selected landmark is illustrated in Fig. 1. Each researcher performed the extraction three times and calculated the mean coordinate value of the original landmark (LMK_Org). Next, the centre of gravity of the original model was moved to the origin of the global coordinate system, and the data was saved in a.obj file.

### Determining SRP

#### Initial alignment of the original and mirror model

For all 15 case models in this study, the original model (Model_Org) was initially superimposed onto its YZ-plane mirror model to obtain an optimal weight distribution of the 32 PA landmarks. Geomagic Studio 2013 software was used for the global ICP registration function. During the process, the original model was fixed, and the mirror model was floated. The mirror model (Model_Mir) was obtained following superimposition, and the corresponding initial mirror landmarks were then established (LMK_Mir).

#### Test group_1: Determining SRP using PA algorithm

The three-dimensional coordinates of all landmarks in the original and mirror images (LMK_Org and LMK_Mir; 32 pairs of landmarks in total) were derived and entered into the PA algorithm program, which was based on the Python language, without weight differences. The transformation matrix of the mirror model was then calculated and loaded onto the Model_Mir using Geomagic Studio 2013. Finally, the SRP of the facial data for each patient was constructed by taking the union of the original and mirror models (Model Uni_PA) in Geomagic Studio using the function ‘‘plane’’ and “symmetry”, defined as ‘SRP_PA’.

#### Test group_2: Determining SRP using WPA algorithm

Similarly, the three-dimensional coordinates of all landmarks in the original and mirror images (LMK_Org and LMK_Mir; 32 pairs of landmarks in total) were derived and entered into the WPA algorithm program, which was based on the Python language. The weight factor for each landmark was automatically calculated based on the distance of paired landmarks. For example, a landmark pair with good symmetry would be relatively close together post initial registration and would thus be given more weight. Conversely, a landmark pair with poor symmetry would be relatively far apart and would thus be given less weight.

The weighted landmarks of LMK_Org and LMK_Mir were superimposed three-dimensionally based on the least-weighted squares, so that optimal superimposition was obtained for the 32 pairs of landmarks and the WPA transformation matrix of the mirror model (Model_Mir) was derived. The transformation matrix was loaded onto the Model_Mir using Geomagic Studio 2013. Finally, the SRP of facial data for each patient was constructed by taking the union of the original and mirror models (Model Uni_WPA), the same procedure as test group_1, defined as ‘SRP_WPA’.

#### Reference group: Determining the ground truth

Studies have shown that the alignment of the original and mirror models for SRP abstraction, based on areas defined by experts having good symmetry, exhibits sufficient adaptability for facial asymmetry cases, but the reliance on expert definitions reduced the degree of algorithm automation. The SRP of an algorithm based on professional expertise and empirical data was regarded as the ground truth in this study. Regions with good facial symmetry from the original and mirror models (Model_Org and Model_Mir) were manually selected by senior doctors using Geomagic Studio software, and regional registration was conducted with the two models (Model_Org fixed and Model_Mir floated). Finally, the SRP of the facial data for each patient was constructed by taking the union of the original and mirror models (Model Uni_Ref). These SRPs were defined as the ground truth (‘SRP_Ref’).

**Fig. 2:**
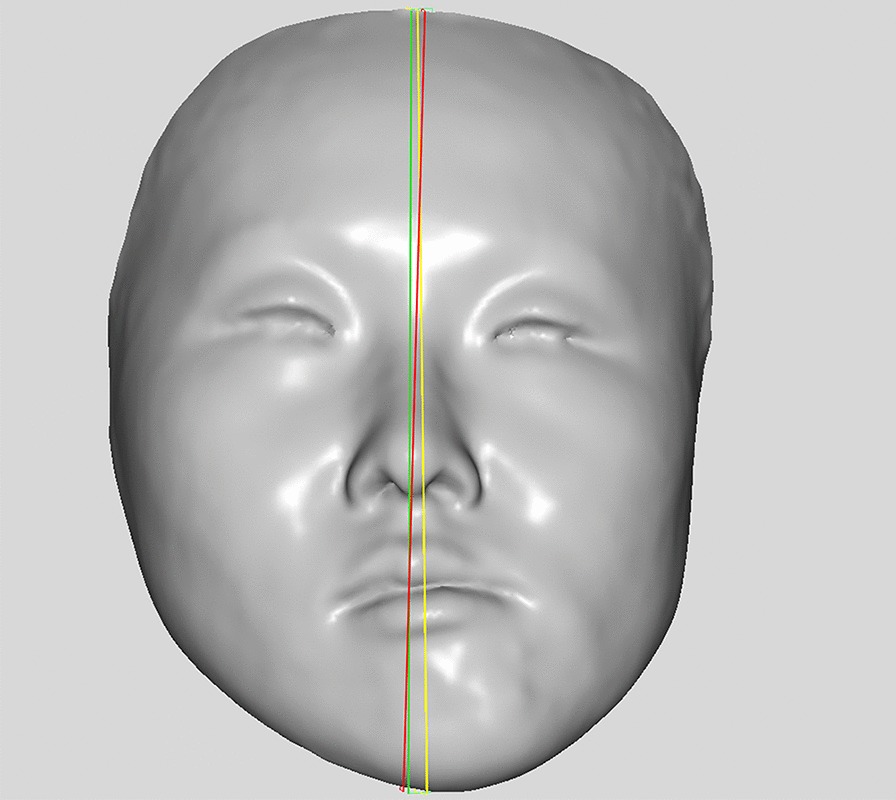
Determining the SRP based on WPA algorithm, PA algorithm and professional algorithm for one case. Red plane signifies SRP based on professional algorithm, green plane represents WPA algorithm, and yellow plane represents PA algorithm

The SRPs constructed using the WPA, PA, and professional algorithms are shown in Fig. 2.

### SRP measurement evaluation

#### Angle error of planes

For each of the 15 three-dimensional mandibular deviation models, the angles between SRP_PA and SRP_Ref and between SRP_WPA and SRP_Ref were calculated and recorded as Err_Ang_PA and Err_Ang_WPA, respectively. The average and standard deviation of the angle error for each sample were also calculated.

#### Position error of the mirrored landmarks

The position error of the mirrored landmarks was defined as a new quantitative index to evaluate SRP, which may further validate the result of the weighted landmarks. The position error indicator was designed to obtain the weight distribution of the WPA algorithm landmarks and professional landmarks (implied empirical information) by calculating the distance between corresponding landmarks in the WPA and professional algorithms. If the two weights are consistent, then the mirror landmark overlap is suitable, and the position error is small. Conversely, if the weights are inconsistent, then the position error is large. The mean value of the position error reflects the consistency between the SRPs of the WPA and professional algorithms in accounting for the weight distribution of the global facial landmarks.

**Fig. 3:**
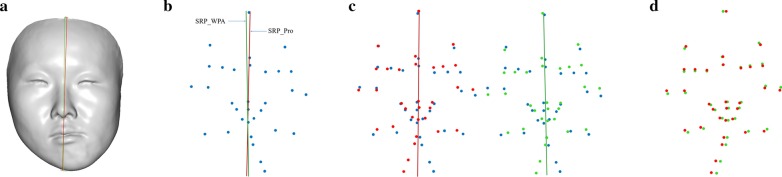
Position error of the mirrored landmarks. **a** SRPs on original three-dimensional face, red colour plane signifies reference plane (SRP_Ref) and green colour represents WPA algorithm plane (SRP_WPA). **b** Blue landmarks signify original landmarks. **c** Reference mirror landmarks in red and WPA mirror landmarks in green, which were obtained from mirror original landmarks using SRP_Ref and SRP_WPA. **d** Global position error was defined as the average distance of the 32 pairs of reference and WPA mirrored landmarks

The mirror landmarks of each model (LMK_PA and LMK_WPA) were obtained from the mirror and original models using the SRP_PA and SRP_WPA. The mirror landmarks of the reference group (LMK_Ref) were similarly obtained. The global position error was defined as the average distance of the 32 landmarks pairs in LMK_PA and LMK_Ref and in LMK_WPA and LMK_Ref. During this process, the original model was fixed in the test and reference groups. The closer each mirror landmark constructed by the SRPs of the test groups was to the same landmark in the reference group (i.e. the smaller the position error), the closer the SRP to the reference plane. The global position error was calculated based on 32 paired landmarks (Err_LMK_WPA and Err_LMK_PA) (Fig. 3).

Huang has shown that facial asymmetry is more obvious in the lower face than upper face [10]. For mandibular deviation patients, the degree of landmarks asymmetry in the lower part of the face is significantly higher than those in the middle and upper parts. Therefore, the weight distribution of features in different regions should differ and cannot be analysed with the global position error. Thus, we also evaluated the regional position error of the three facial partitions. The regional position error was calculated for landmarks in each facial third partitions: 4 landmarks in the upper third, 17 in the middle third, and 11 in the lower third, named Err_LMK_WPA_Up and Err_LMK _PA_Up, Err_LMK_WPA_Mid and Err_LMK _PA_Mid, and Err_LMK_WPA_Low and Err_LMK _PA_Low, respectively. The average and standard deviation of the global and regional position error were calculated for each sample.

#### FAI error

The FAI error was calculated based on the SRP constructed for the test groups of the 15 facial data and defined as the sum of the distance from the medical landmark to the SRP and the difference between bilateral landmarks and the SRP. FAI_PA, FAI_WPA, and FAI_Ref were obtained according to formula 4. Err_FAI_WPA and Err_FAI_PA were defined as the difference between the FAI values of the WPA and professional algorithm and the difference between the FAI values of the PA and professional algorithms, respectively. The average value and standard deviation of the FAI error of each sample were calculated.

4 $$FAI = \mathop \sum \limits_{i = 1}^{10} Md_{i} + \mathop \sum \limits_{i = 1}^{11} \left| {Rd_{i} - Ld_{i} } \right|$$

*Md*_*i*_ represents the distance from the medical landmark to the SRP. *Rd*_*i*_ and *Ld*_*i*_ represent the differences between the right landmark and the SRP, and that between the left landmark and the SRP, respectively.

### Statistical analysis

Statistical analysis was conducted using SPSS software (Version 21, SPSS Inc., Chicago, IL, USA). A K-S normality test was conducted for the angle error (of two groups), the global position error (of two groups), the regional position error (of six groups), and the FAI error (of two groups) to assess data distribution (15 calculated values per group).

**Fig. 4:**
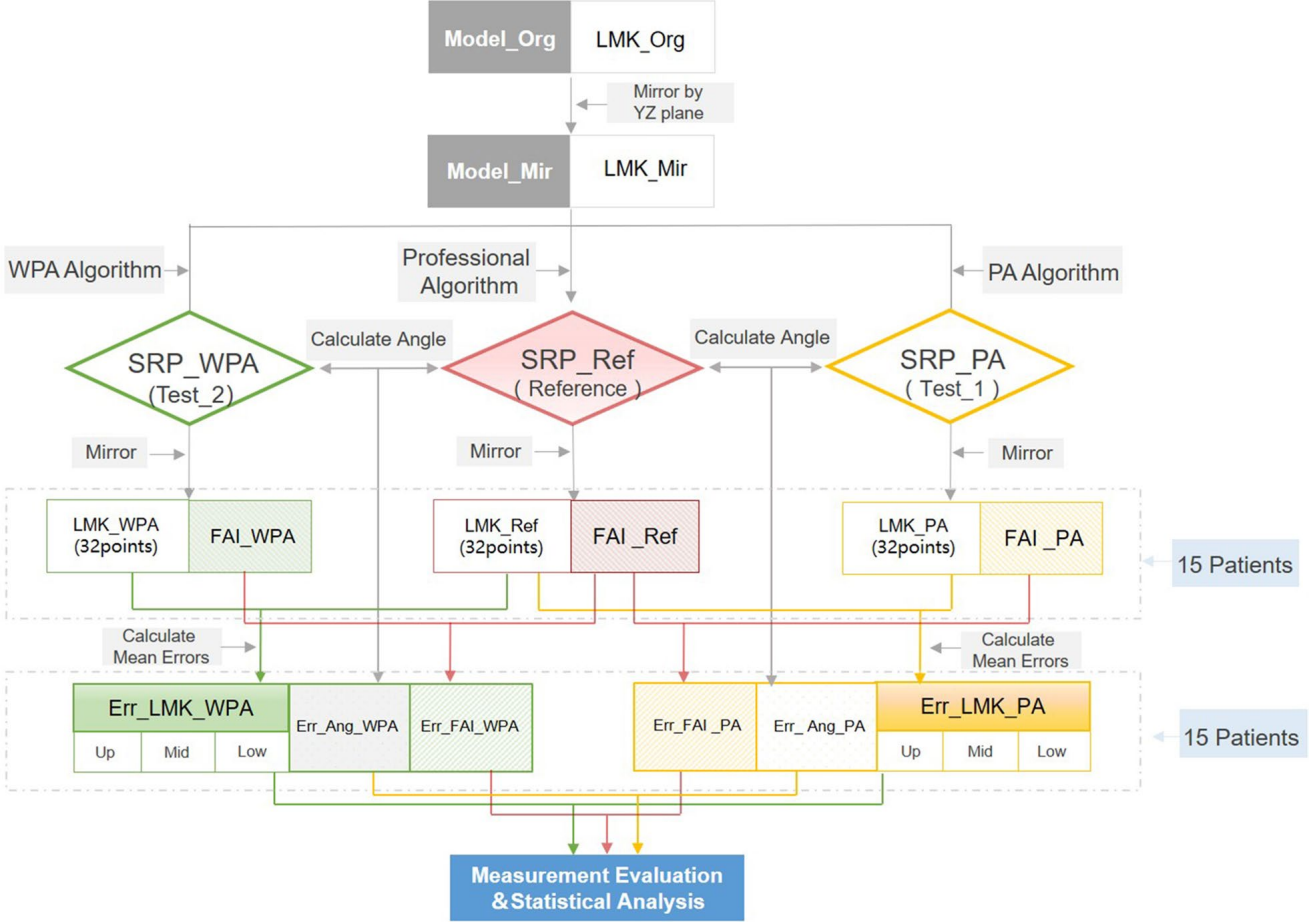
Workflow of the experimental procedures and evaluation methods. In the figure, WPA represents Weighted Procrustes analysis algorithm, PA represents Procrustes analysis algorithm. SRP_WPA、SRP_PA and SRP_Ref are symmetry reference planes constructed by WPA group, PA group and reference group respectively, LMK_WPA, LMK_PA and LMK_Ref are mirror landmarks constructed by WPA algorithm, PA algorithm and professional algorithm symmetry reference plane. FAI_WPA, FAI_PA and FAI_Ref are the facial asymmetry index (FAI) calculated by the SRP defined by WPA algorithm, PA algorithm and professional algorithm, Err_LMK_WPA and Err_LMK_PA are the global landmarks position errors of WPA and PA algorithms, under which Up, Mid and Low represent the position errors of different third parts. Err_Ang_WPA and Err_Ang_PA are the angle errors of WPA algorithm and PA algorithm. Err_FAI_WPA and Err_FAI_PA are facial asymmetry index (FAI) errors of WPA algorithm and PA algorithm

The workflow of the experimental procedures and evaluation methods are shown in Fig. 4. We performed a paired *t* test analysis of the position error of both the WPA and PA algorithm groups of 15 patients to evaluate the overlapping differences of the WPA and PA algorithms in terms of global and regional landmarks. A statistical significance was set at *P* < 0.05.

A one-way ANOVA analysis was performed on regional landmarks of position error to examine whether differences in the position error of different facial partitions were statistically significant. A homogeneity-of-variance test was also performed. Tukey’s honesty significance test was used for multiple comparisons. A paired *t* test analysis was also conducted to compare angle and FAI errors.

## Results

### Analysis of angle error

The K–S normality test for angle error (of two groups of 15 values each) showed that both groups conformed to the normal distribution. Data analysis yielded no significant differences (*P* > 0.05) between the PA and WPA algorithm groups. Measurement analysis showed that the mean and standard deviation of the angle error in the PA and WPA groups were 2.16 ± 1.08° and 1.66 ± 0.81°, respectively. Since the mean and standard deviation of the angle error of the WPA algorithm group were smaller, this indicates that the SRP constructed using the WPA algorithm for the 15 data points was closer to the ground truth plane.

### Analysis of position error

Table 1 shows the measurement values for the position error between the test groups for global landmarks (two groups of 15 values each) and regional landmarks (six groups of 15 values each). The K–S normality test for position error revealed that all groups conformed to the normal distribution. There were significant differences in the position errors among the groups (*P* < 0.05).

**Table 1:** The position error of global and regional facial landmarks (upper, middle, lower) (mm)

Subject	Err_LMK (mm)		Err_LMK_Up (mm)		Err_LMK_Mid (mm)		Err_LMK_Low (mm)	
No	WPA	PA	WPA	PA	WPA	PA	WPA	PA
1	4.50	5.18	4.71	5.78	3.70	4.00	5.23	6.21
2	4.59	5.59	1.94	2.99	4.07	5.32	6.21	11.97
3	1.53	3.35	1.08	6.01	1.53	3.16	1.68	2.66
4	5.22	5.95	3.08	4.50	4.16	4.64	7.62	8.52
5	3.14	3.43	3.36	2.03	3.45	3.34	2.59	4.08
6	4.29	5.03	1.99	3.38	4.65	4.28	4.58	6.78
7	1.23	2.38	1.40	3.27	0.98	1.85	1.56	2.89
8	2.75	5.08	1.37	4.12	2.59	3.97	3.50	7.15
9	4.29	4.24	2.57	2.42	3.39	3.34	6.30	6.28
10	5.02	8.87	2.16	7.37	4.60	7.11	6.70	12.15
11	6.77	7.35	4.60	5.55	5.74	5.78	9.13	10.42
12	3.09	2.66	2.66	2.28	2.93	2.26	3.50	3.43
13	1.95	1.85	0.94	0.92	1.77	1.69	2.60	2.45
14	3.76	4.21	2.08	2.71	3.03	3.44	5.51	5.96
15	2.42	2.93	1.74	1.73	2.38	2.32	2.73	4.31
Mean ± SD	3.64 ± 1.53	4.54 ± 1.92	2.38 ± 1.15	3.67 ± 1.84	3.27 ± 1.29	3.77 ± 1.51	4.63 ± 2.28	6.35 ± 3.23

**Fig. 5:**
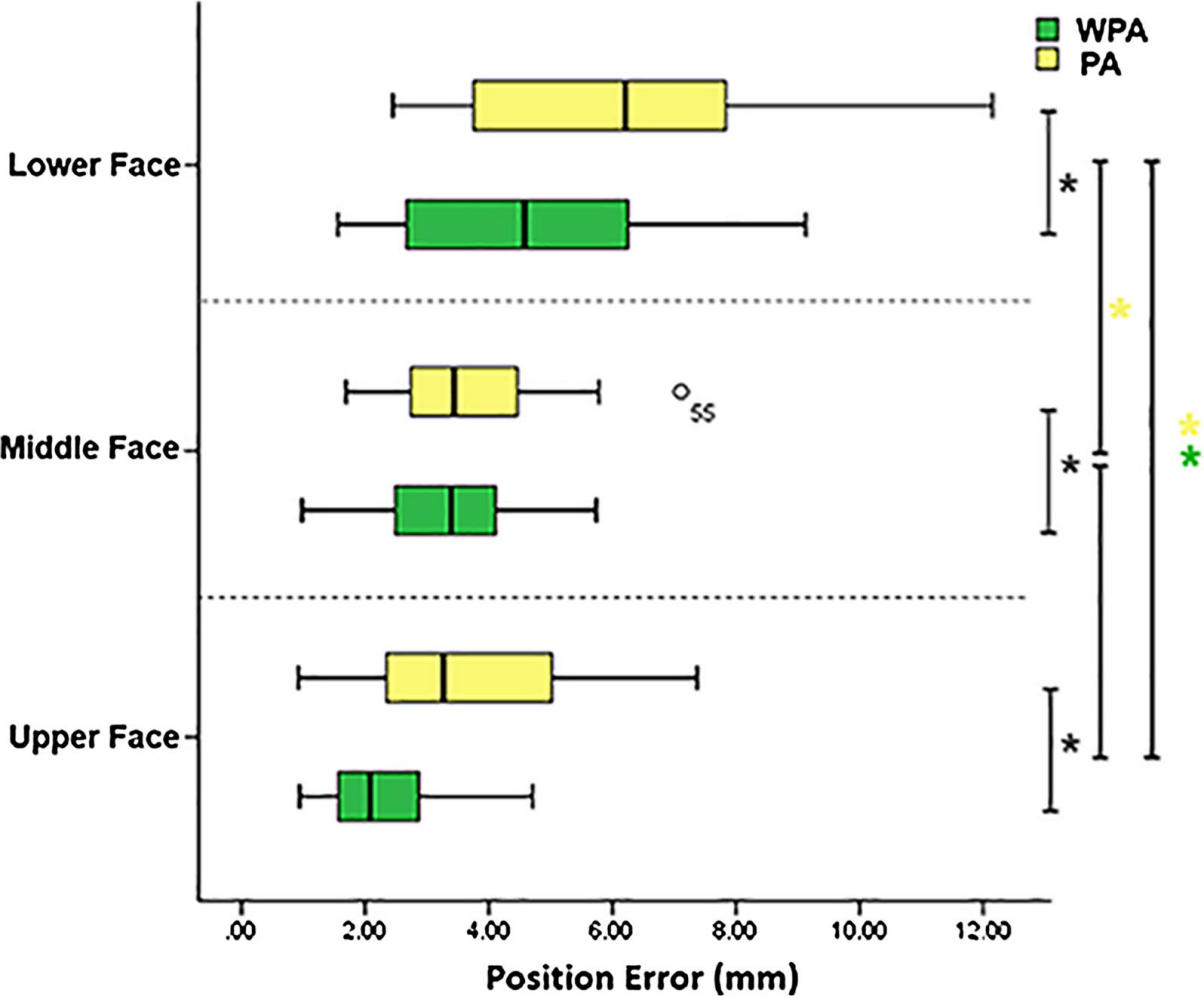
Boxplot of position error for upper face, middle face, lower face group. The black asterisks signify *P* < 0.05 between WPA algorithm and PA algorithm group. The yellow and green asterisks indicate statistical significance for position error of different regional groups using a one-way ANOVA followed by Tukey’s multiple comparison test where *P* < 0.05, the circles within the boxplot represent outliers

Tukey’s honesty significance test revealed significant differences (*P* < 0.05) between the lower and upper facial partitions in the WPA group, between the lower and upper partitions in the PA group, and between the lower and middle partitions in the PA group. Related sample data distribution and statistical analysis results are shown in Fig. 5.

The mean and standard deviation of the global position error of the WPA and PA groups were 3.64 ± 1.53 mm and 4.54 ± 1.92 mm, respectively; the mean and standard deviation of the position error of the WPA algorithm group were smaller than those of the PA group. Among the six groups of regional facial data, the position errors of the upper, middle, and lower partitions in the WPA group were 2.38 ± 1.15 mm, 3.27 mm ± 1.29 mm, and 4.63 ± 2.28 mm, respectively; the position error was lowest in the upper partition. The difference between the lower partition error and the global mean position error was 0.99 mm.

In the PA group, the position errors of the upper, middle, and lower partitions were 3.67 ± 1.84 mm, 3.77 ± 1.51 mm, and 6.35 ± 3.23 mm, respectively; the position error was highest in the lower partition. The difference between the lower partition error and the global mean position error was 1.81 mm. These results showed that the global and regional errors of the WPA group were smaller than those of the PA group. Additionally, the lower partitions weighted overlap result of the WPA group was closer than that of the PA group to the weighted overlap result of the reference group, demonstrating a significant improvement in the WPA group compared with the PA group.

### Analysis of FAI error

The K–S normality test for FAI error (in two groups of 15 values each) showed that both groups conformed to the normal distribution. Data analysis yielded no significant differences (*P* > 0.05) between the PA and WPA groups. There were no significant differences between the FAI errors of both groups (*P* > 0.05). Measurement analysis showed that the average FAI errors calculated with the WPA and PA algorithms were 13.65 ± 12.45 mm and 15.77 ± 14.32 mm, respectively. The result of the WPA-calculated SRP was closer to the SRP of the ground truth plane than the PA-calculated SRP.

## Discussion

### The WPA SRP was more closely aligned with the ground truth plane than the standard PA SRP

The weighted algorithm is an important innovation of this study. The degree of the landmarks symmetry could be evaluated quantitatively and used as landmark weight factors to construct an SRP. Our WPA algorithm is designed to assign a small weight for landmarks with poor symmetry, post initial global ICP superimposition of the original and mirror models, and a large weight for landmarks with good symmetry. The weight calculation method was based on the reciprocal of the distance between the paired landmarks, which represents an inverse relationship between the distance and the corresponding assigned weight. Based on superimposition using least-weighted squares, all original and mirror PA landmarks were assigned different weights. The solution to the PA landmark set system (the WPA objective function) was minimised, thus achieving an optimal overlap result of the original and mirror landmarks.

The results indicated that the average angle error of WPA group for all enrolled patients with mandibular deviation was < 2°, although there was no significant result when compared with the average angle error of the standard unweighted PA algorithm (of which the error was > 2°), the result of the WPA SRP was closer to the ground truth (Fig. 2), and the angle error displayed a downward trend.

Wu et al. showed that the angle difference between the two planes is easily perceived when it is > 6° [23]. The angle error between the WPA SRP and the reference plane was < 2°, which indicates that the accuracy of the WPA SRP was almost equal to that of the reference plane and therefore had a better clinical suitability than the PA SRP. Furthermore, the stability level of the WPA algorithm, with a standard deviation of 0.81°, was significantly higher than that of the PA algorithm, which had a standard deviation of 1.08°.

Additionally, the FAI value calculated for the WPA algorithm was closer to the professional result than was the FAI value calculated for the PA algorithm. Furthermore, the WPA FAI for patients with mandibular deviation was closer to the ground truth plane than the PA FAI. These results confirmed that the WPA algorithm performed better than the PA algorithm in constructing facial SRPs for facial asymmetry (mandibular deviation).

### A new SRP evaluation indicator: the position error of mirror landmarks

In previous SRPs studies of the face and skull, SRP evaluation indicators have primarily included the angle and FAI errors [24, 25]. These two indicators can assess the global proximity of SRP, but neither can quantitatively analyse facial landmark asymmetry. In this study, we proposed to use the position error indicator as a novel SRP evaluation tool.

The mirror landmarks differed between the test and reference SRPs for mirroring the original facial model, while the original model was the same between the test and reference groups. Table 1 indicates that the mean values of the global position errors of the WPA and PA algorithms were 3.64 mm and 4.54 mm, respectively, and that the difference between them was statistically significant. This indicates that the global overlapping degree of the WPA algorithm mirrored features and reference mirrored features was more accurate than that of the PA algorithm. The weight distribution of the WPA algorithm was also significantly more accurate than that of the PA algorithm; the weight factor of the WPA algorithm had a significant effect.

The mean value of the regional position error for the upper, middle, and lower partitions also reflected the degree of consistency between the weight distribution of the WPA SRP and the reference SRP for each facial partition. The mean position error of the WPA algorithm was smaller than that of the PA algorithm for all three facial partitions. This difference was significant, indicating that the WPA algorithm for each facial partition was close to the professional algorithm.

Additionally, the position error of the WPA algorithm for the upper and lower parts of the face was considerably smaller than that of the PA algorithm, while that for the middle part was close to that of the PA algorithm. This is because the WPA algorithm allocated a lower weight for lower facial landmarks to reduce their influence on the global overlapping degree, while the upper landmarks were assigned higher weights to increase the overlapping degree, thus accounting for professional experience in the weight distribution of the landmarks. Compared with the PA algorithm without weight distribution, the position error of the WPA in each region was optimised, and an ideal SRP construction result was obtained.

### Limitations and further research to improve the three-dimensional facial SRP

Previous studies on the original-mirror alignment method are divided with regards to using the ICP and PA algorithms. Among them, ICP is an algorithm that does not refer to anatomical landmarks. Although the reliability and repeatability of the ICP algorithm have verified when used for constructing SRPs with data from patients with normal facial symmetry, facial asymmetry data affects algorithm’s performance making SRPs construction unfeasible for patients with severe asymmetry. Scholars have since improved the global ICP algorithm by manually selecting facial regions with good symmetry for original and mirror models; the clinical suitability of this modified ICP algorithm has improved to some extent [26, 27]. This algorithm is referred to as the regional ICP algorithm, and although it reduces the degree of automation by introducing human interference, it remains suitable for use in oral clinics. Therefore, the regional ICP algorithm was used as the ground truth in this study to evaluate the accuracy of our proposed algorithm.

One of the differences between the PA and ICP algorithms is that SRP extraction using the PA algorithm relies more on anatomical facial landmarks, which is consistent with clinical diagnosis and treatment. PA algorithm is applicable for symmetry patients, but the asymmetric PA landmarks will have a Pinocchio effect on the PA algorithm [28].

One source of improvement is to filter PA landmarks. Landmarks have been sorted through the recursive PA algorithm, deleting the obvious asymmetric landmarks (outliers) and using the remaining for PA operation to avoid their interference [29]. However, for patients with complex facial deformities (in which most landmark symmetries are not ideal), this algorithm may eliminate too many landmarks and tends to be locally over-optimised.

Our study has proposed another way to improve the standard PA algorithm by adding a weighted system. We hypothesised that by analysing the distance between the corresponding original and mirror landmarks post initial alignment, the degree of symmetry could be evaluated quantitatively and used as landmark weight factors to construct an SRP with personalised feature weight assignments. Our WPA algorithm did not have a reduced degree of automation and could therefore simulate the expression of the reference value weight of anatomical landmarks assigned according to clinical experience. This is advantageous with regards to SRP construction. Our results also indicated that the WPA algorithm was suitable for patients with complex mandibular deviation. However, the WPA algorithm tested in this study had some limitations.

First, the quantitative indicator of landmarks asymmetry (the reciprocal of the distance between paired landmarks) was indirectly obtained. To set the key parameters for the landmark weight factors, global ICP algorithm was used to initiate the registration of the original and mirror models. One-way to address this is to use an intelligent landmark weighting strategy based on direct morphological feature analysis, artificial intelligence, and deep learning technology, to improve the accuracy and rationality of landmark weight distribution leading to better SRP constructions that simulate expert clinical diagnosis.

Second, anatomical landmarks in this study need to be selected manually. We expect that our WPA algorithm will further combine mathematical facial mask, automatically extracting a general face mesh, thus improving its clinical suitability. Cases of mandibular deviation between 5 and 23 mm were quantitatively analysed in this study; although sample cases should be further expanded to evaluate our method’s suitability for different types and degrees of facial deformities to provide guideline for clinical application. Therefore, testing our method on samples representing a wider range of facial deformities is warranted.

## Conclusion

The WPA SRP was more closely aligned than the standard PA SRP with the ground truth plane in terms of angle and FAI errors as well as global and regional position error, indicating that our novel method of assigning weights to facial landmarks had accurately constructed an SRP for patients with facial asymmetry. We also established the position error as an effective SRP analysis tool for facial asymmetry data. Our proposed method and findings can help stomatological clinical practices in both mandibular deviation diagnosis and treatment. In addition, this new method is not restricted to three-dimensional facial data and can be applied to skeletal models providing new solutions for dental clinical practise.

## Data Availability

The datasets used and analyzed during the current study are available from the corresponding author on reasonable request.

## Abbreviations

CCD: Charge-coupled device
FAI: Facial asymmetry index
FH: Frankfort horizontal
ICP: Iterative closest point
PA: Procrustes analysis
SRP: Symmetry reference plane
WPA: Weighted Procrustes analysis
